# The effect of toxic pyridine-alkaloid secondary metabolites on the sunbird gut microbiome

**DOI:** 10.1038/s41522-020-00161-9

**Published:** 2020-11-13

**Authors:** Mohanraj Gunasekaran, Maya Lalzar, Yehonatan Sharaby, Ido Izhaki, Malka Halpern

**Affiliations:** 1 Department of Evolutionary and Environmental Biology, Faculty of Natural Sciences, University of Haifa, Mount Carmel, Haifa Israel; 2 Bioinformatics Service Unit, University of Haifa, Mount Carmel, Haifa Israel; 3 Department of Biology and Environment, Faculty of Natural Sciences, University of Haifa, Oranim, Tivon Israel

**Keywords:** Microbiome, Microbial ecology

## Abstract

Sunbirds feed on tobacco tree nectar which contains toxic nicotine and anabasine secondary metabolites. Our aim was to understand the effect of nicotine and anabasine on the gut microbiota composition of sunbirds. Sixteen captive sunbirds were randomly assigned to two diets: artificial nectar either with (treatment) or without (control) added nicotine and anabasine. Excreta were collected at 0, 2, 4 and 7 weeks of treatment and samples were processed for bacterial culture and high-throughput amplicon sequencing of the 16S rRNA gene. The gut microbiome diversity of the treated and control birds changed differently along the seven-week experiment. While the diversity decreased in the control group along the first three samplings (0, 2 and 4 weeks), it increased in the treatment group. The microbiota composition analyses demonstrated that a diet with nicotine and anabasine, significantly changed the birds’ gut microbiota composition compared to the control birds. The abundance of nicotine- and anabasine- degrading bacteria in the excreta of the treated birds, was significantly higher after four and seven weeks compared to the control group. Furthermore, analysis of culturable isolates, including *Lactococcus*, showed that sunbirds’ gut-associated bacteria were capable of degrading nicotine and anabasine, consistent with their hypothesised role as detoxifying and nutritional symbionts.

## Introduction

The guts of animals harbour complex microbial communities that are important for physiology, immune system development, nutrition and detoxification reactions in their hosts^1–3^. The majority of the studies on microbiomes have focused on human and husbandry hosts^4^. Studies on gut-microbiome in avian hosts have been largely overlooked^3^. Of the limited number of studies on avian gut microbiomes, most were carried out on domestic birds like chickens, and turkeys^3^ rather than on wild birds. Furthermore, only a few studies have examined the gut bacteria of passerine birds^5–10^. As far as we know, there are only three studies on gut microbiota of nectarivorous birds; (i) nitrogen-recycling in the gut of Anna’s hummingbirds (*Calypte anna*)^11^, (ii) gut microbiota composition of the rufous-tailed hummingbird, *(Amazilia tzacatl*)^9^ and (iii) comparison between the microbial communities on bills and excreta of Anna’s hummingbirds and black-chinned hummingbirds (*Archilochus alexandri*) and their food resources (feeders and floral nectar).^10^

The importance of specific bacteria for digestive recycling in avian species with large ceca and well-developed gastrointestinal microbiotas has been documented^12^; however, most nectarivorous and frugivorous birds have only vestigial ceca. For example, hummingbirds, arguably the most specialized avian nectarivores, have no ceca and perform extremely fast digestion throughout the entire digestive tract, which may limit colonization by bacteria^3,13,14^. Therefore, it has been assumed that the gastrointestinal tracts of birds that feed on nectar do not have the structures needed to house extensive microbiota, as is presumably required for effective digestive recycling^3^.

Here we report on an experimental study based on the natural relationship between the tobacco tree (*Nicotiana glauca*), and its Old-World nectar consumer, the orange-tufted sunbird (*Cinnyris osea*). *N. glauca* is native to Argentina and Bolivia and is also found in other parts of South America, California, Hawaii, Africa, Australia and in the Mediterranean region, including Israel^15^. The pollination of *N. glauca* is dependent upon pollinating vectors because its stamens are shorter than the stigma^16^. Because it has relatively long corolla, *N. glauca* mainly depends on birds with long bills, such as sunbirds and hummingbirds, for pollination^15^. The orange-tufted sunbird is a small passerine bird, weighing 6–7 g, that inhabits parts of the Middle East and Sub-Saharan Africa. It has a long, slender, decurved bill (1.4–2.0 cm in length) with a long tongue, which allows it to feed mainly on floral nectar^17^. Like other nectarivores, the sunbirds feed on carbohydrate-rich foods with low-protein content and have high sugar-absorption efficiencies despite the rapid speed that food moves through their gut^18^. Consequently, sunbirds supplement their diets with arthropods in order to meet the nutritional requirements of their nestlings^19^. In Israel, sunbirds are the main pollinators of *N. glauca* (60% are legitimate visitors that feed on nectar from the front of the flower)^20^.

The nectar of *N. glauca* is rich in sugar with a mean sugar equivalent concentration of 20 ± 0.3% (mean ± SEM). It contains the toxic pyridine alkaloids nicotine and anabasine at concentrations of 0.50 ± 0.12 ppm and 5.0 ± 0.8 ppm (means ± SEM), respectively^20,21^. The widespread existence of ‘toxic nectar’, or nectar with secondary metabolites, is puzzling given that one of the most crucial functions of floral nectar is to attract mutualists, such as legitimate pollinators^22^. Nevertheless, plant secondary metabolites may play an adaptive role as mediators of mutual plant-animal interactions, such as pollination and seed dispersal, and thus may increase plant fitness^23–25^. The ‘direct toxicity hypothesis’ suggests that plant secondary metabolites control or filter out nectar robbers and allow only the appropriate pollinators to feed on nectar^26,27^.

We hypothesized that plant secondary metabolites in nectar mediate the interactions between plants and their nectar consumers by shaping the gut microbiome of the latter. Specifically, we aim to understand (i) the effect of pyridine alkaloids in nectar on gut microbiota composition of nectarivores and (ii) whether the gut microbiota of nectarivores contains bacteria that can degrade plant pyridine alkaloids. To accomplish these goals, we conducted feeding experiments to study how natural concentrations of nicotine and anabasine that are found in the tobacco tree nectar, affect the microbiome of the orange-tufted sunbird, that uses the plant’s nectar as a food source and acts as one of its pollinators.

## Results

### Sunbirds feeding experiment

**Fig. 1:**
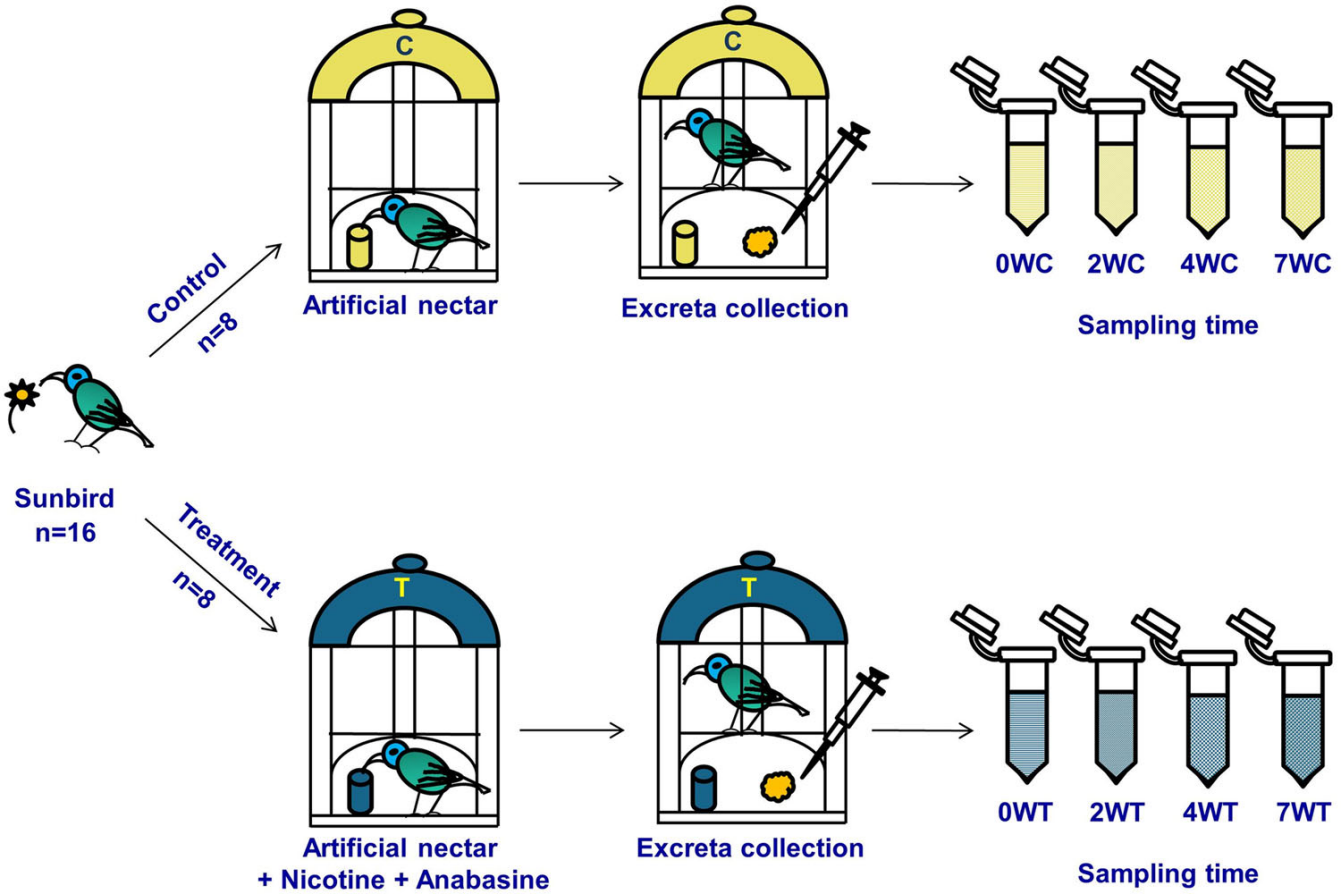
Feeding experiment setup. Sixteen naive sunbirds were captured and divided into two groups: (i) a control group that was fed with artificial sunbird nectar free of alkaloids and (ii) a treatment group that was fed with artificial nectar supplemented with nicotine (0.5 ppm) and anabasine (5 ppm). Excreta samples were collected from both groups at day 0 and the end of weeks 2, 4 and 7. All samples were used for generating 16S rRNA libraries using Illumina sequencing and for bacterial community analyses. Excreta that were collected from the birds at day 0 and at 4 weeks were used for culturing bacterial isolates that can degrade nicotine or anabasine. W sampling week, C control, T treatment.

Sixteen orange-tufted sunbirds were captured and adapted to laboratory conditions for a period of 4 weeks. Then, birds were randomly divided into two groups by sex, such that each group was comprised of four males and four females. The control group was fed the artificial nectar without any additional nutrients while the treatment group was fed the same artificial nectar with the addition of nicotine and anabasine (0.5 ppm and 5 ppm, respectively: Fig. 1). Excreta were collected from each of the 16 birds on day 0 and at the end of weeks 2, 4 and 7 (Fig. 1). The excreta samples were used for culture-independent and culture-dependent bacterial analyses (see ‘Methods’). All results are presented as the mean ± standard error of the mean (SEM).

### Culture-independent results

To study the effect of diet and sampling week on bacterial community composition and diversity, we analysed the microbiota of sunbird excreta using sequence data obtained by amplicon sequencing of 16S rRNA gene fragments. Overall, 278 amplicon sequence variants (ASVs), were detected across the entire dataset. The rarefaction curves of each sample reached an asymptotic level (Supplementary Fig. 1), suggesting that our sampling efforts were sufficient to obtain a full estimate of ASV richness.

### The effect of diet and sampling week on gut-microbiome composition and diversity

**Fig. 2:**
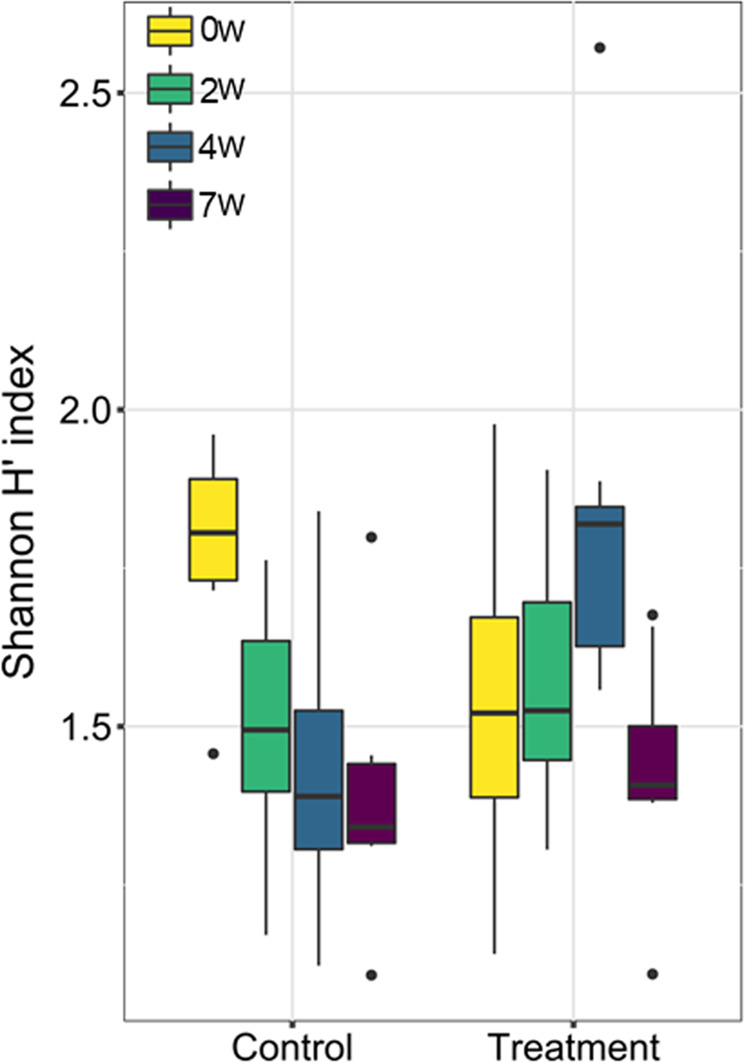
Alpha diversity (Shannon H′ index) of bacterial communities in sunbird excreta at the different sampling times with respect to diet (control vs. treatment). The figure demonstrates that the gut microbiome diversity of the treated and control birds changed differently along the seven sampling weeks. Whereas diversity decreased in the control group in the 2nd, 4th and 7th week samples, it increased in the treatment group. The diet vs. week was significantly different (*F* = 17.1, df = 3, *p* < 0.001). See also Supplementary Table 1. Results are presented as the mean ± SEM.

To examine effects of diet and sampling week on bacterial diversity, samples were rarefied to equal read depths (7500 sequences per sample) and Shannon H’ index was calculated. Shannon H′ diversity suggested a change over time in excreta bacterial community composition, which was more pronounced in control compared with treated birds (Fig. 2). The most pronounced effects were observed after 4 weeks in both treated and control sunbirds. Nonparametric analysis of longitudinal data, using a nested design confirmed significant effects of the sampling week (*F* = 15.7, df = 3, *p* < 0.01) and week × diet interaction (*F* = 17.1, df = 3, *p* < 0.001), indicating that the two groups demonstrated different diversity patterns throughout the experiment (Fig. 2). No direct effect of diet (control vs. treatment) was observed (*F* = 2.21, df = 1, *p* = 0.14). Moreover, no direct effects of sex nor its interaction with either sampling week or diet were observed (*p* > 0.05, Supplementary Table 1).

**Fig. 3:**
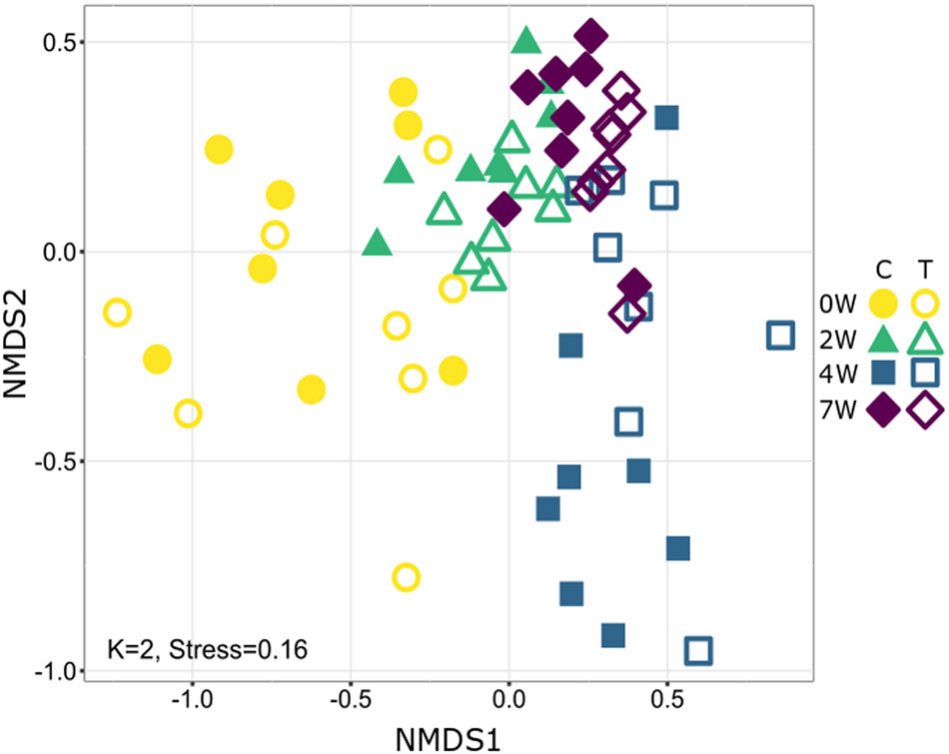
Non-metric multidimensional scaling analysis (NMDS) based on the Bray–Curtis dissimilarity matrix. This visualization demonstrates the dynamic changes in the gut microbiota composition of sunbirds from the different feeding groups: the control group (C) with no addition of secondary metabolites and the treatment group with the addition of nicotine and anabasine (T). Each group contained 8 birds that were held in separate cages. Excreta samples were collected at day 0 (0 W) and after 2 (2 W), 4 (4 W) and 7 (7 W) weeks.

To visualize the effects of diet and sampling week on microbiota composition, non-metric multidimensional scaling (NMDS) was performed (Bray–Curtis dissimilarity matrix, *K* = 2, stress = 0.16), demonstrating a time-dependent effect of diet (control vs. treatment) on the bacterial community composition (Fig. 3). Furthermore, microbiota composition, tested using a permutational analysis of variance (PERMANOVA) on a Bray–Curtis dissimilarity matrix, was significantly affected by sampling week (*F* = 14.79, df = 3, *R*^2^ = 0.38, *p* < 0.05) and also by diet, though the effect was smaller (*F* = 3.9, df = 3, *R*^2^ = 0.03, *p* < 0.05) (Table 1). In this model, interactions among variables were marginal. Overall, the factors examined explained 63% of the variance (residuals = 0.27, Table 1).

**Table 1:** The effect of nicotine and anabasine diet on sunbird gut microbiota composition (based on 16S rRNA amplicon sequencing).

Factor	DF	F model	*R*^2^	*P* value	FDR adj. *p* value
Week	3	14.79	0.38	0.001	0.013
Diet	1	3.89	0.03	0.003	0.039
Sex	1	2.46	0.02	0.028	0.364
Diet × Bird	2	2.79	0.05	0.003	0.039
Week × Diet	3	2.26	0.06	0.006	0.078
Week × Sex	3	1.68	0.04	0.041	0.533
Diet × Sex	1	0.97	0.01	0.428	1.00
Week × Diet × Sex	6	0.94	0.05	0.560	1.00
Week × Diet × Sex	3	0.67	0.02	0.842	1.00
Diet × Bird × Sex	2	1.01	0.02	0.415	1.00
Week × Diet × Sex × Bird	6	0.94	0.05	0.584	1.00
Residuals	32		0.27		
Total	63		1.00		

Results of permutational analysis of variance (PERMANOVA) based on the Bray–Curtis dissimilarity matrix. The nested factorial model included diet (C control, T treatment), bird identifier (as a nested factor within treatment), sampling week (samples taken at day 0 and after 2, 4 and 7 weeks), sex and their interactions.

### Taxa composition in the sunbird gut

**Fig. 4:**
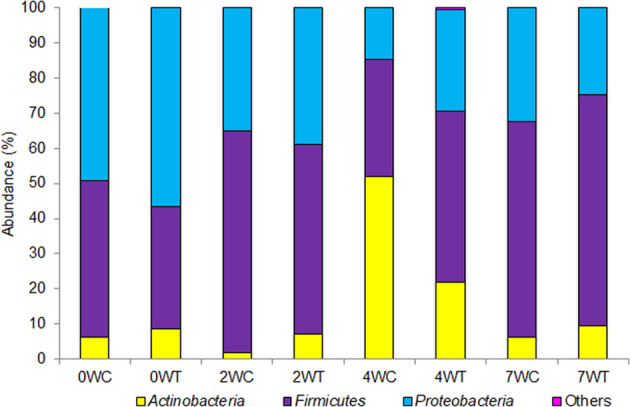
Mean relative abundances of different bacterial phyla in the control and the treatment groups. W sampling week, C control, T treatment.

*Proteobacteria*, *Firmicutes* and *Actinobacteria* were the three dominant bacterial phyla detected in the experimental samples (Fig. 4). In the naive birds (at day 0), *Proteobacteria* was the dominant phylum (49.20 ± 6.24% in control and 56.54 ± 4.82% in the treatment samples), but it decreased thereafter with time. In order to identify the bacterial phyla and specific amplicon sequence variants (ASVs) that varied most between the control and treated birds, we utilized the linear discriminant analysis (LDA) effect size (LEfSe) method and compared excreta bacterial community composition after 2, 4 and 7 weeks. After two weeks, *Firmicutes* became dominant in both the control (63.23 ± 3.33%) and treatment (53.77 ± 3.61%) groups. At this time point, *Actinobacteria* contributed significantly to the dissimilarities between the groups, with a higher abundance of 7.21 ± 1.58% in the treatment compared to 1.73 ± 0.69% in the control (*p* = 0.0012; Fig. 4). At the end of the fourth week, *Proteobacteria* were significantly more abundant in the treatment group (29.05 ± 3.28%) compared to the control group (14.71 ± 4.05%, *p* = 0.049). By contrast, *Actinobacteria*, which were less abundant in both groups in the previous time intervals, showed the highest prevalence at week four and were now significantly more abundant in the control compared to the treatment group (52.03 ± 7.58 and 21.90 ± 6.1%, respectively, *p* = 0.037). *Firmicutes* was still the most dominant phylum in the treatment group (48.39 ± 4.71%); however, their abundance did not vary significantly compared to the control group (33.10 ± 8.12%; Fig. 4). After seven weeks, *Firmicutes* was the most dominant phylum in both the control (61.10 ± 4.55%) and the treatment groups (65.80 ± 5.30%), followed by *Proteobacteria*, with a relative abundance of 32.52 ± 3.35% in the control and 24.67 ± 2.50% in the treatment groups (Fig. 4). *Bacteroidetes* was the phylum which contributed most to dissimilarities between the groups at this stage even though their abundance was relatively low, with 0.005 ± 0.004% in the control compared to 0.07 ± 0.04% in the treatment (*p* = 0.017).

**Fig. 5:**
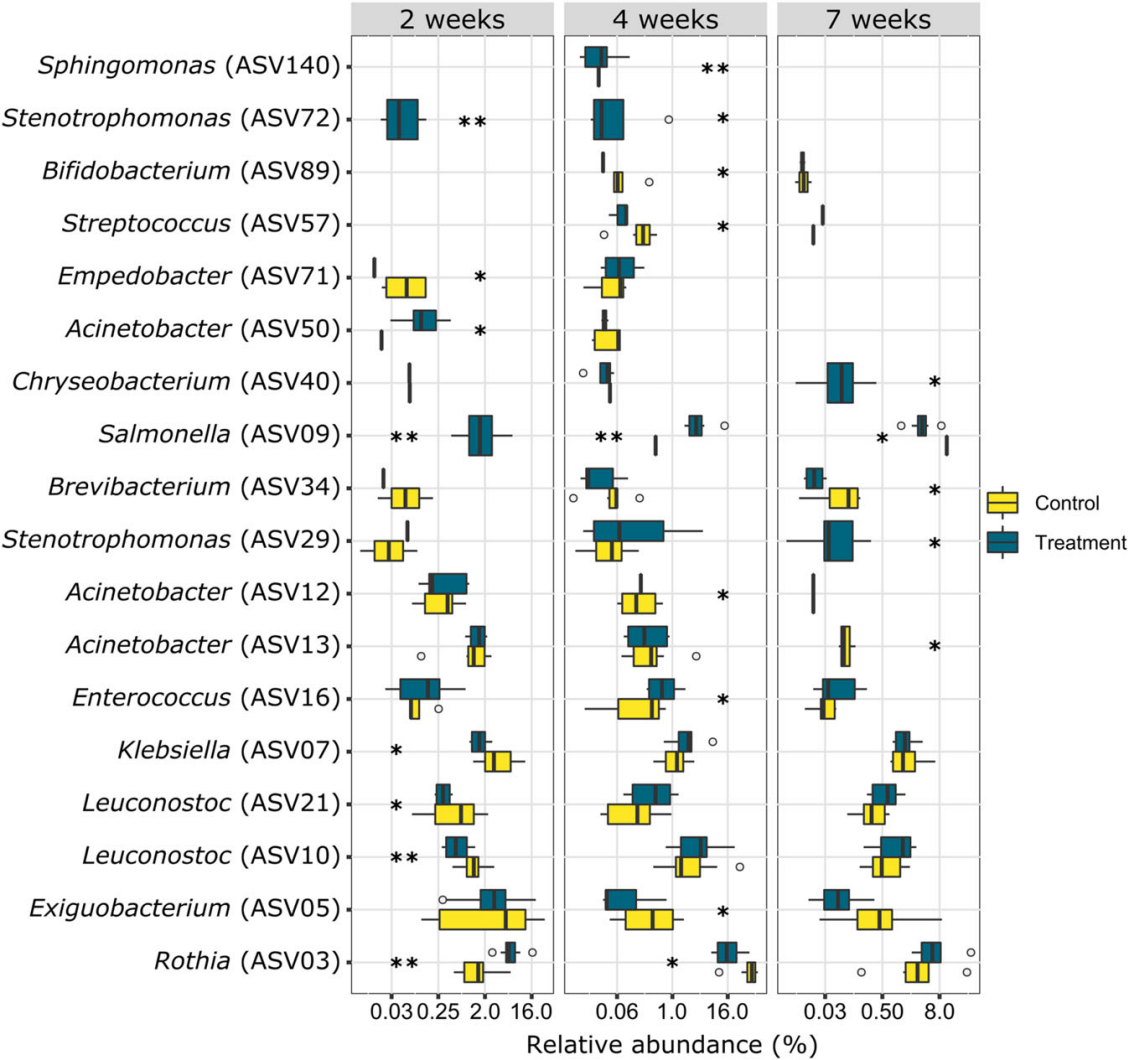
Biomarker ASVs of control vs. treated birds at 2, 4 and 7 weeks. ASVs with significant differences confirmed by linear discriminant analysis (LDA) effect size (LEfSe) (*P* < 0.05, LDA effect size > 2.0) are presented. Asterisks denote significant differences (*p < 0.05, **p < 0.01). Results are presented as the mean ± SEM.

At the clonal level, a total of 18 ASVs were found to be significantly affected by diet in at least one of the sampled weeks, for example: *Acinetobacter (*ASV 13) at week 7; *Exiguobacterium* (ASV 5) at week 4; *Rothia* (ASV 3) at weeks 2 and 4; *Salmonella* (ASV 9) at weeks 2, 4 and 7; *Stenotrophomonas* (ASV 72) at weeks 2 and 4 (Fig. 5, Supplementary Table 2).

### Culture-dependent survey and bacterial function

A total of 146 nicotine/anabasine-degrading strains were isolated from sunbird excreta and identified on minimal media that contained nicotine or anabasine as the sole carbon and nitrogen sources. At day zero, 28 isolates were identified across all 16 birds. After four weeks, 118 isolates were identified (Table 2), of which 51 were isolated from the control birds and 67 from the treated birds. The isolates belonged to 6 bacterial classes (five represented in the control group and six in the treatment group) and 14 genera. Most of the isolates at day zero belonged to the *Gammaproteobacteria* class and to four species in the genus *Enterobacter* (Table 2).

**Table 2:** Nicotine- and anabasine-degrading bacterial isolates.

Class	The closest species identity in the GenBank database	0 Weeks	4 Weeks	
		0WC, 0WT	4WC	4WT
		*n* = 16 birds	*n* = 8 birds	*n* = 8 birds
*Alphaproteobacteria*				
	*Methylorubrum thiocyanatum*	–	–	1 (98.5)
	*Methylorubrum populi*	–	–	1 (99.0)
	*Roseomonas mucosa*	–	–	1 (99.6)
*Gammaproteobacteria*				
	*Pseudomonas geniculata*	1 (95.0)	–	–
	*Pseudomonas aeruginosa*	3 (96.7)	11 (99.0–100.0)	17 (99.0–100.0)
	*Pseudomonas hibiscicola*	–	–	1 (96.4)
	*Delftia lacustris*	–	1 (99.00)	–
	*Acinetobacter pittii*	8 (96.0–100.0)	1 (99.8)	3 (99.0–99.9)
	*Acinetobacter junii*	1 (99.0)	–	2 (99.0–99.8)
	*Acinetobacter bereziniae*	–	3 (99.0–99.7)	–
	*Enterobacter hormaechei subsp. oharae*	2 (96.3)	–	–
	*Enterobacter mori* (*tabaci*)	7 (98.0–99.0)	–	–
	*Enterobacter hormaechei subsp. xiangfangensis*	2 (98.0–99.6)	7 (99.1–99.5)	10 (99.1–99.2)
	*Enterobacter hormaechei subsp. hormaechei*	–	2 (99.0–100.0)	1 (100.0)
	*Enterobacter ludwigii*	1 (98.6)	–	–
	*Stenotrophomonas pavanii*	–	2 (99.4–99.5)	2 (99.1–99.3)
	*Stenotrophomonas rhizophila*	–	3 (97.0–99.3)	3 (97.4–99.1)
	*Stenotrophomonas maltophilia*	–	2 (99.5–99.7)	–
	*Klebsiella michiganensis*	–	1 (98.7)	1 (99.4)
	*Klebsiella quasipneumoniae subsp. similipneumoniae*	–	1 (99.0)	–
	*Klebsiella grimontii*	–	3 (98.7–99.6)	4 (98.6–99.4)
*Flavobacteriia*				
	*Chryseobacterium gleum*	2 (99.1–99.2)	5 (99.2–99.6)	2 (98.6–99.4)
*Bacilli*				
	*Exiguobacterium indicum*	1 (99.5)	2 (99.0–99.5)	2 (99.1–99.5)
	*Lactococcus lactis subsp. hordniae*	–	–	1 (99.6)
*Actinobacteria*				
	*Brevibacterium sanguinis*	–	4 (98.0–99.6)	1 (99.4)
	*Kocuria palustris*	–	2 (97.9–98.5)	
*Sphingobacteriia*				
	*Sphingobacterium spiritivorum*	–	–	13 (98.5–99.8)
	*Sphingobacterium multivorum*	-	1 (98.5)	1 (98.9)

Isolates were cultured at time 0 before nicotine and anabasine were added to the birds’ artificial diet (0WC, 0WT) and 4 weeks after starting the experiment (4WC and 4WT). Isolates were identified by sequencing their 16S rRNA genes (GenBank accession numbers MK348690–MK348835). Numbers before the parenthesis indicate the number of isolates. Numbers in parenthesis represent percentage of identity to type strain species. See also Supplementary Table 4.

### Putative functional link

Interestingly, all isolates were able to degrade both nicotine and anabasine, although they were enriched and isolated on only one kind of medium (M9 with nicotine or M9 with anabasine; Table 2).

**Fig. 6:**
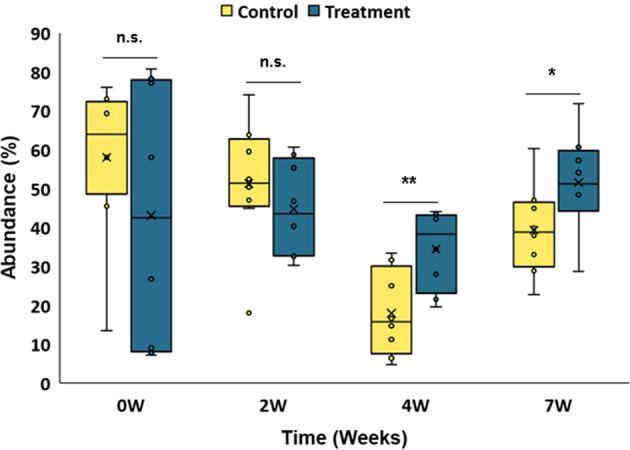
Relative abundance of nicotine- and anabasine-degrading bacteria at each sampling week in control (*n* = 8) and treated (*n* = 8) birds. X marks group means. n.s., not significant. Asterisks indicate significant differences at the *p < 0.05 and **p < 0.01, according to independent sample *t*-tests (one-tailed hypothesis, arcsine transformed abundance). Results are presented as the mean ± SEM.

We analysed the dynamics of the genera which were identified in the excreta and also classified as nicotine-degrading bacteria in the literature or which were isolated as nicotine-and anabasine-degrading species in the current study (Table 2). To verify whether the ASVs’ sequences coincided with the 16S rRNA genes that were generated to identify the culturable isolates, we compared the sequences and found a 100% match between corresponding ASV and the isolate sequences (Supplementary Table 3). Screening the entire dataset yielded a total of 24 bacterial genera (Supplementary Table 4). The relative abundances of those genera were summed for each sample and compared between the control and treatment groups (Fig. 6). As expected, there was no significant difference in the abundance of nicotine-degrading genera between the groups at time zero (57.96 ± 7.27% in the control and 43.08 ± 11.94% in the treatment group, *t* = 1.094, df = 11.3, *p* = 0.15, Fig. 6); however, in the treatment group their abundance increased to 51.50 ± 4.53% after seven weeks while the opposite was observed in the control group, where the average abundance of the same bacterial genera eventually decreased to 39.31 ± 4.13% (Fig. 6). The relative abundance of nicotine-degrading bacteria was significantly higher in the treatment group compared to the control in both the fourth and seventh weeks (fourth week: *t* = 3.09, df = 14, *p* = 0.004; seventh week: *t* = 1.98, df = 14, *p* = 0.034).

## Discussion

Birds’ gut microbiomes are increasingly important model systems from an ecological and evolutionary perspective because of their relevance to host fitness, longevity, disease resistance and adaptation^28^. Here we explored the effect of alkaloids produced in the nectar of *N. glauca* on the gut microbiota of *C. osea*. We observed significant differences between the bacterial communities assembled in control versus treated birds’ excreta (Fig. 3 and Table 1) supporting the hypothesis that the presence of secondary metabolites in nectar shapes the gut microbiome of nectarivores.

Diet affects not only the microbiota composition but also its diversity. Our results showed that the gut microbiota diversity of the treated and control birds changed differently along seven sampling weeks. For example, whereas sunbird gut microbiota diversity decreased in the control group along the first three samplings (0, 2, 4 weeks), it increased in the treatment group (Fig. 2). The gut microbiota diversity of the treated birds increased until the fourth week and then decreased after seven weeks (Fig. 2). Pyridine alkaloids may stimulate both positive and negative responses in various bacteria. Hence, the increased diversity of the gut microbiota of the treatment group during the first four weeks may reflect a transitional state of the gut microbiota composition, in which the suppressed bacteria are still present alongside the appearance of new bacterial species. Such transition in gut microbiota diversity during feeding experiments was also observed in chickens that were fed a calcium-enriched diet compared to chickens that were fed a control diet^29^. However, a partial explanation for gut microbiota dynamics could be a continued stress effect of capturing and handling wild-caught sunbirds and feeding them an artificial diet, although the bird in our study were acclimated for four weeks to captivity conditions. The stress may gradually affect physiology, immunity, metabolism and consequently their normal (wild) gut microbiomes.

*Proteobacteria*, *Firmicutes* and *Actinobacteria* were relatively abundant phyla in the sunbirds’ gut microbiota in both the control and the treatment groups at all sampling intervals (Fig. 4). Similarly, these were also the most abundant phyla in hummingbird gut microbiota^10^ as well as in neotropical birds^30^ and in vampire ground finch (*Geospiza difficilis septentrionalis*)^31^. A different pattern, though, was described by Waite and Taylor^32^ who found that *Proteobacteria, Firmicutes* and *Bacteroidetes* were the most abundant phyla in avian gut microbiota.

In a recent study, Aizenberg et al.^21^ studied the influence of nicotine on the bacterial community composition of nectar of wild-type *N. glauca* and wild-type and transgenic *N. attenuata*. They^21^ found that the elimination of nicotine from the transgenic *N. attenuata* (in which nicotine biosynthesis was silenced), significantly affected the bacterial community of the nectar compared to the wild-type plants. Although the bacterial community composition in *N. glauca* nectar was different from that of *N. attenuata*, both in its wild-type and transgenic forms, the two wild-type plants that contained nicotine in their nectar were more similar when compared to the manipulated transgenic *N. attenuata* plant. Thus, nicotine manipulation in *Nicotiana* plants affects the microbiota composition of the floral nectar^21^. It, therefore, seems that pyridine alkaloids have a general effect on microbiota composition of various hosts, and our findings support this.

Nicotine can be toxic even in a relatively low concentration, and at the same time, this metabolite can be the source of nutrients if enzymes that can degrade it are present. Here we demonstrated that nicotine and anabasine, when added to artificial nectar in concentrations in which they naturally occur in floral nectar, affect the sunbirds’ gut microbiota composition and diversity, and shape the gut microbiome of these nectarivorous birds.

Remarkably, all the isolates in the current study (Table 2) degraded both nicotine and anabasine, although they were enriched and isolated on only one kind of pyridine alkaloid. Nicotine and anabasine are structurally similar isomer alkaloids found in *N. glauca* nectar. Anabasine was previously reported as being more toxic to animals and humans than nicotine^33^. Bacterial degradation of nicotine and anabasine shares common pathway processes like dehydrogenation and hydroxylation. Cross-competencies, the ability to degrade and catabolize both these alkaloids, have been previously observed and studied in several bacterial genera such as *Pseudomonas* and *Arthrobacter*^34^. However, the unique difference observed between these two alkaloids is the dehydrogenation that occurs before hydroxylation in the case of nicotine degradation whereas during anabasine degradation, the hydroxylation occurs first and is then followed by dehydrogenation^35^.

In nature, birds consume plant products and invertebrates that contain secondary metabolites that are toxic when absorbed^36^. According to Dearing et al.^37^, birds may harbour bacteria that can detoxify these toxic metabolites. For instance, plant saponins were found to be degraded by bacteria in the crops of hoatzins (*Opisthocomus hoazin*)^38^, and several mycotoxins were metabolized by bacteria associated with the chicken gut^39^. A caecal metagenome analysis of greater sage grouse (*Centrocercus urophasianus*) revealed that its microbiota was enriched with genes related to the metabolic pathways that can degrade phenols to pyruvates as well as genes responsible for xenobiotic degradation and the metabolism of terpenoids^40^. Kohl et al.^40^ also found genes that were responsible for the biosynthesis of essential amino acids. They suggested that these bacterial genes can assist the host in maintaining nitrogen balance. Nectarivores may have a problem balancing their nitrogen stores because floral nectar contains low levels of proteins^41^. Tsahar et al.^42^ showed that nectar-feeding birds have low nitrogen requirements, but the mechanisms that these animals use to conserve nitrogen remain unclear. Here we suggest that the gut microbiota of sunbirds degrade pyridine alkaloids and thus act as nutritional symbionts which may promote their nitrogen balance.

There are various reports of nicotine degradation by different bacterial genera including *Delftia, Klebsiella, Stenotrophomonas Pseudomonas* and *Lactobacillus*^43–47^. Here we isolated bacteria capable of growing on nicotine and anabasine as sole carbon and nitrogen sources (Table 2). These include isolates identified to five genera, *Chryseobacterium*, *Exiguobacterium*, *Lactococcus*, *Methylorubrum* and *Kocuria*, which, as far as we know, have not previously been reported to degrade nicotine or anabasine. Similarly, Ceja-Navarro et al.^48^ isolated bacteria from the gut of the coffee borer beetle (*Hypothenemus hampei*) that had the ability to degrade the toxic alkaloid caffeine by using it as its sole sources of carbon and nitrogen. The majority of the caffeine-degrading isolates belonged to the genus *Pseudomonas*. In our study, *Pseudomonas* isolates were also a prominent genus among nicotine- and anabasine-degrading isolates (Table 2). Ceja-Navarro et al.^48^ showed that the gut bacterial community of *H. hampei* metabolized and detoxified caffeine and thus promoted the reproduction and fitness of the host.

In our study, we found a significant change in the bacterial community composition, such that a number of genera with the potential ability to degrade the toxic metabolites nicotine and anabasine (Supplementary Table 4) were significantly more abundant after the 4th and the 7th weeks of the experiment in the treated birds compared to the control birds (Fig. 6). During microbial degradation of nicotine, different bacterial species release the end products (like methylamine) to their growth medium^49^. Methylamine is degraded to ammonium by different microorganisms and can be used as a nitrogen source^50^. Thus, these nicotine-degrading bacteria, whose relative abundances increased in the presence of nicotine and anabasine after the 4th and the 7th weeks of treatment, may play important functional roles in the gut, likely with direct benefits to their hosts.

However, there is an alternative hypothesis that sunbirds may host only transient, opportunistic, N-limited parasite bacteria which were consumed with the nectar^9,51^. If this is the case, the alkaloid-degrading bacteria in their guts are not symbionts which supply a mutualistic service of detoxification but rather transients. Another alternative hypothesis is that alkaloids change the microbiomes of the treatment diet, and those differences were then maintained during passage through the sunbirds’ guts and thus amplified in their excreta. The detoxification and nutritional symbiosis hypothesis has yet to be robustly tested; future work could make use of antibiotic-treated birds or metabolic labelling using alkaloids incorporating nitrogen stable isotope N^15^.

## Methods

### Studied organisms

We studied the microbiota composition of orange-tufted sunbirds (*Cinnyris osea*) that feed on the tobacco tree *N. glauca* nectar. In Israel, sunbirds are the main pollinators of *N. glauca*^15^.

### Ethical statement

All methods were performed in accordance with relevant guidelines and regulations. Sunbirds were captured in Israel with the permission of the Israel Nature and Parks Authority (permit #2016/41432). All experimental procedures and animal care were approved by the Committee of Animal Experimentation of the University of Haifa (permit #477/16, expiration date September 2020). In total, 16 adult sunbirds were captured between December 2017 and January 2018 with mist nets and held in captivity for about 12 weeks. Each bird was held in a separate cage in a room with controlled temperature (25 °C) and 12 h:12 h light:dark conditions. After the experiment ended, the birds were set free.

### Sunbird feeding experiment

After capture, the 16 birds were acclimated to laboratory conditions (see above) and fed artificial nectar (Sunbird nectar special formula for Nectariniidae; Aves & Avian, Lot nr IS240718; Reg.nr. NL113333, Raalte, Netherlands) and water, for a period of 4 weeks.

After 4 weeks of acclimation, the birds were grouped by sex, and then each sex was randomly divided into two groups. The control group (eight birds, four males and four females) was fed the artificial nectar mentioned above, without additional nutrients. The treatment group (eight birds, four males and four females) was fed the same artificial nectar with the addition of nicotine and anabasine (Sigma Aldrich, Rehovot, Israel), in concentrations that naturally occur in *N. glauca* (0.5 ppm and 5 ppm, respectively^20^; Fig. 1). Fresh artificial nectar and water were supplied to both groups, daily.

Excreta were collected from each of the 16 birds on day zero (before adding nicotine and anabasine to the treatment group) and at the end of weeks 2, 4 and 7 (Fig. 1). The excreta collection procedure was as follows: a new, clean piece of baking paper was spread on the bottom of each cage so that the excreta would not be contaminated by the cage surface; once the bird left its excreta, it was immediately collected using a sterile pipette tip into a sterile Eppendorf tube.

The excreta samples were used for DNA extraction for microbiota composition analyses (culture-independent) and to isolate nicotine/anabasine-degrading bacteria, as described below.

### Culture-independent approach

Excreta samples for culture-independent analyses were kept at –20 °C until DNA was extracted using a DNeasy Blood and Tissue isolation kit (Qiagen, Hilden, Germany), according to the manufacturer’s instructions. DNA was also extracted from three blank samples without addition of excreta.

The genomic DNA was PCR-amplified using primers targeting the V4 region of the 16S rRNA gene. The primers were: CS1_515F (ACACTGACGACATGGTTCTACAGTGCCAGCMGCCGCGGTAA) and CS2_806R (TACGGTAGCAGAGACTTGGTCTGGACTACHVGG-GTWTCTAAT). Primers were synthesized by Sigma Aldrich (Rehovot, Israel) and contained 5′ common sequence tags^52^. The amplification was performed in 25 µl reaction volumes using Emerald Amp MAX HS PCR Master Mix (Takara Bio Inc., Otsu, Shiga, Japan). Primer concentrations were 0.5 ng/µl. The PCR conditions were as follows: 95 °C for 5 min, followed by 28 cycles of 30 s at 95 °C, 45 s at 55 °C, and 30 s at 68 °C. A final elongation step of 7 min at 68 °C was included. The amplification products were verified by agarose gel electrophoresis and then stored at −20 °C. DNA control extractions (without excreta) did not produce bands on agarose gels after PCR amplification with the primers. These samples were not sequenced.

Before sequencing the samples, a second PCR amplification was performed in a 10 μl reaction in a 96-well plate. The master mix used for the reaction was made using 2X AccuPrime SuperMix II (Thermo Fisher Scientific, Massachusetts, United States). A final concentration of 400 nM of each primer was used, and each respective well in the 96 wells plate received a separate primer set with a unique 10-base barcode (Fluidigm, South San Francisco, CA, USA; item #100-4876). The unique barcodes in separate reactions were used for the positive control and a second no-template control reaction with only Access Array Barcode library primers was also run. The amplification conditions were 95 °C for 5 min, followed by 8 cycles at 95 °C for 30 s, 60 °C for 30 s and 68 °C for 30 s. A final, 7 min elongation step was performed at 68 °C. The amplified products of positive and negative controls and selected samples were validated using Qubit fluorometric quantitation with the Qubit 2.0 Fluorometer (Life Technologies, Carlsbad, CA, United States). After determining the quality of amplification, the samples were collected in equal volume and purified in solid phase reversible immobilization (SPRI). The final quality control was performed using Agilent 2200 TapeStation and Qubit analysis, prior to dilution to 6 pM for emulsion PCR. Pooled, diluted libraries were pair-ends sequenced on an Illumina MiSeq instrument and analysed with Casava 1.8 using pipeline 1.8 (Illumina, San Diego, CA, USA). The reads were 250 nucleotides in length and PhiX DNA served as a spike-in control. Barcode sequences from Fluidigm were provided to the MiSeq server, and sequences were automatically binned according to their 10-base multiplex identifier sequences. Raw reads were recovered as FASTQ files. The second PCR amplification and Illumina MiSeq sequencing were performed at the DNA Services Facility, University of Illinois, Chicago, USA.

Sequence data were analysed using the DADA2 pipeline^53^. FASTQ-formatted reads were trimmed and filtered for low quality using the command ‘filterAndTrim’ with parameters maxEE = 2, maxN = 0, trimleft = 15 and trunclen = 150. Error rate estimation was carried out using the ‘learnerror’ command with default parameters, except for the randomize parameter, which was set to TRUE, in order to sample nucleotides and reads for model building randomly across all samples. Following these steps, the DADA2 algorithm was implemented for error correction, and a count table containing the amplicon sequence variants (ASVs) and counts per sample was produced. Suspected chimeras were detected and removed using the command ‘removeBimeraDenovo’ with default parameters. Count tables with ASV sequences and the number of reads per sample were extracted. For taxonomy assignment, ASV sequences were aligned using BLASTn to NCBI’s nt database. BLAST results were analysed by the latest common ancestor (LCA) algorithm in MEGAN (version 6.18.1)^54^ with parameters min score >100, max expected <1.0E^−13^ and top percent <1. ASVs assigned to non-bacterial domains, as well as those assigned to mitochondria or chloroplast were removed. In total, 3,543,227 quality bacterial sequences were obtained for the 64 excreta samples of sunbirds (mean = 55,363 ± 13,454), clustered into 278 ASVs.

Raw sequence data were submitted to the National Center for Biotechnology Information (NCBI) Sequence Read Archive (https://www.ncbi.nlm.nih.gov/bioproject/) under the BioProject accession number PRJNA548382.

### Culture-dependent approach

The nicotine/anabasine-degrading bacteria were isolated at two different sampling time points: (i) from excreta collected from all naive birds at day 0, (0WC, *n* = 8; 0WT, *n* = 8), hereafter, “weeks control” and “weeks treatment” are WC and WT, respectively; and (ii) from excreta collected from all birds four weeks after starting the experiment (4WC and 4WT; Fig. 1). The excreta were collected as described above. Collected samples were immediately cultured. To enrich nicotine- or anabasine-degrading bacteria, we used M9 minimal medium (Na_2_HPO_4_.7H_2_O–64 g, KH_2_PO_4_–15 g, NaCl–2.5 g, H_2_O–1 L), sterilized by autoclaving. To 200 ml of this mixture, we added 700 ml water, 2 ml sterile 1 M MgSO_4_ and 100 µl 1 M CaCl_2_, and the whole solution was adjusted to 1 L by adding H_2_O along with the addition of either 0.1% nicotine or 0.1% anabasine as the only carbon and nitrogen sources. Samples of 100 µl of the collected excreta were incubated in 500 µl of M9 minimal medium at 37 °C for 3–6 h. After this enrichment incubation, samples were inoculated on agar plates with the same medium but with the addition of 2% agar. Plates were incubated at 37 °C for 5–7 days.

Nicotine- and anabasine-degrading bacterial colonies were picked and streaked five times on Luria-Bertani (LB, HiMedia Laboratories, Mumbai, India) agar plates. Their ability to grow on nicotine or anabasine as the only carbon and nitrogen sources was verified again by growing them on M9 agar plates with nicotine or anabasine as carbon and nitrogen sources. Pure bacterial isolates were store in LB broth with 30% glycerol at –80 °C.

The bacterial isolates were identified by amplifying a 1501-bp internal fragment of the 16S rRNA gene, in accordance with Senderovich et al.^55^. Purified PCR products were sequenced at MCLAB (South San Francisco, CA, USA) and analyses of all sequences were carried out using the EzTaxon website (http://eztaxon-e.ezbiocloud.net/)^56^. The sequences were deposited in the GenBank database under the accession numbers: MK348690–MK348835.

### Statistical analysis

All statistical analyses were performed in R version 3.6.3^57^ unless otherwise specified. In order to estimate the efficiency of sequencing depth in representing excreta microbiota diversity, we performed rarefaction analysis using the iNEXT package^58^. Rarefaction curves indicated that the depth of sampling was sufficient. For calculation and comparison of alpha-diversity parameters, due to differences in sample sizes, the count matrix was rarefied to the minimum sequence depth (7500 sequences per sample) using the Vegan package^59^ command ‘rarefy’. Following this, the number of observed ASVs, as well as the Shannon H′ alpha diversity index, were calculated using Vegan R package command ‘diversity’. The effects of treatment and sampling week on excreta microbiota diversity (Shannon H′ index) were tested using nonparametric analysis of longitudinal data (npaLD) designed for factorial experiments^60^ using npaLD R package command ‘npaLD’ with diet (control vs. treatment) and sampling week as the model factors and bird identifier as the subject (alpha index ~ week × treatment, subject = bird).

Variation in microbiota composition (beta diversity) in excreta samples was explored using non-metric multidimensional scaling (NMDS). For this purpose, count data (not rarefied) were Hellinger normalized using the Vegan ‘decostand’ command. NMDS was performed with Vegan command ‘metaMDS’ with parameters distance = ’bray’, k = 2, try = 1000 and autotransform = FALSE. Further, we examined the contribution of sampling week, diet, sex and their interactions to variation in microbiota composition. This was done using permutation-based analyses of variance (PERMANOVA) implemented with the Vegan function ‘ADONIS’ with a nested model design (week × diet/bird × sex). Bray–Curtis dissimilarities calculated from Hellinger-transformed counts data were used for the ADONIS tests, and the Bonferroni multiple hypothesis testing correction method (FDR) was applied to the results (Table 1).

To identify which phyla and ASVs contribute significantly to variation in microbiota due to treatment, we used the linear discriminant analysis (LDA) effect size (LEfSe) method^61^. For this purpose, count data was normalized by the cumulative sum of squares method (CSS^62^) with R package metagenomSeq^63^. LefSE was performed with CSS normalized counts to compare treatment and control groups for weeks 2, 4 and 7, using the online tool Galaxy (version 1.0; http://huttenhower.sph.harvard.edu/galaxy/) with default parameters but without the counts per million transformation. The threshold for the logarithmic LDA score for discriminative features chosen was >2.0. LefSE results are presented in Supplementary Table 2.

We calculated and compared the change in abundance of potential nicotine and anabasine degraders within the bacterial communities in the excreta along the seven experimental weeks between the control and the treatment groups (Supplementary Table 4). Their relative abundances were summed for each sample and compared between the control and treatment groups at each sampling week using independent sample, one-tailed t-tests (after arcsine transformation). Variances were not equal between groups at time zero according to Levene’s test (*p* < 0.05), and statistical values were corrected accordingly. All results are presented as the mean ± standard error of the mean (SEM).

### Reporting summary

Further information on research design is available in the Nature Research Reporting Summary linked to this article.

## Supplementary information

Supplementary Information

Reporting Summary

## Data Availability

The sequence data from this study are available under the BioProject accession number PRJNA548382 in National Center for Biotechnology Information (NCBI) Sequence Read Archive (https://www.ncbi.nlm.nih.gov/bioproject/). The 16S rRNA sequences are available in the GenBank database under the accession numbers: MK348690–MK348835.
