# Supplementary material for: The effect of toxic pyridine-alkaloid secondary metabolites on the sunbird gut microbiome
Source: NPJ Biofilms Microbiomes. 2020 Nov 13;6:53. doi: 10.1038/s41522-020-00161-9 (PMC7666580; doi:10.1038/s41522-020-00161-9)
Supplement: Supplementary file 1 — Supplementary Information [file 41522_2020_161_MOESM1_ESM.pdf]

SUPPLEMENTARY FILE

The effect of toxic pyridine-alkaloid secondary metabolites on the sunbird gut microbiome

Mohanraj Gunasekaran,[1] Maya Lalzar,[2] Yehonatan Sharaby,[1] Ido Izhaki[1] and Malka Halpern[1, 3]

[1]Department of Evolutionary and Environmental Biology, Faculty of Natural Sciences, University of Haifa, Mount Carmel, Haifa, Israel
[2]Bioinformatics Service Unit, University of Haifa, Mount Carmel, Haifa, Israel
[3]Department of Biology and Environment, Faculty of Natural Sciences, University of Haifa, Oranim, Tivon, Israel

Supplementary figure

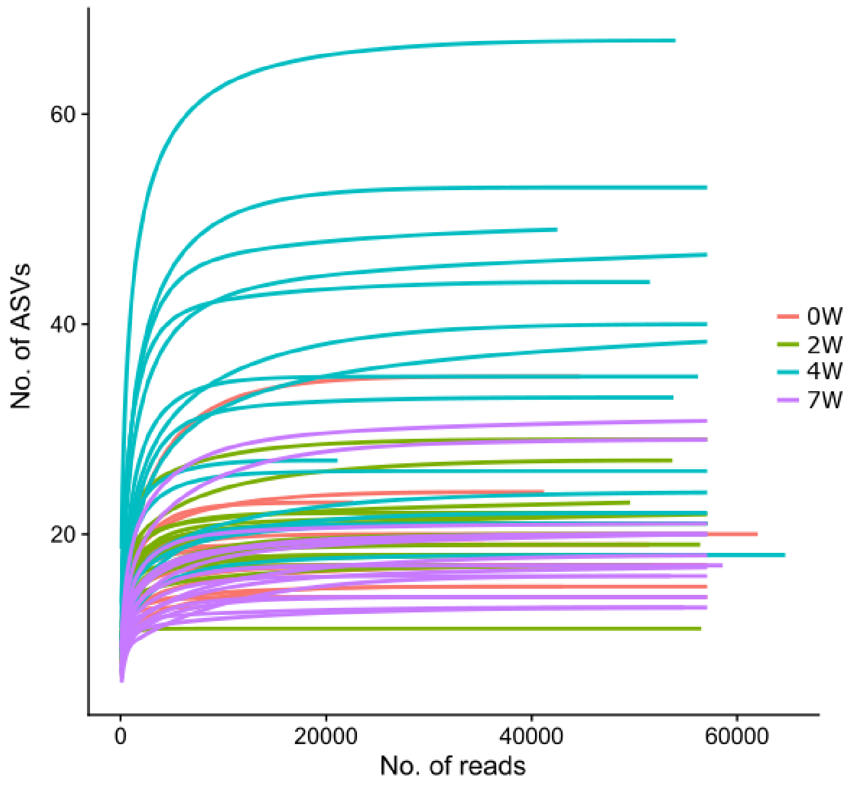

Supplementary Figure 1. Rarefaction curve of the control and treatment samples from the different sampling weeks (day 0 and after 2, 4 and 7 weeks). The curve indicates the estimated number of amplicon sequence variants (ASVs) in all samples at various sampling depths of sequence reads. The rarefaction curves of each sample reached an asymptotic level, suggesting that our sampling efforts were sufficient to obtain a full estimate of ASV richness. W, week

Supplementary Tables

Supplementary Table 1. **Comparison of alpha diversity (Shannon H' index) of bacterial communities in sunbird excreta at the different sampling weeks with respect to diet (control vs. treatment) and sex**. Nonparametric analysis of longitudinal data (npaLD) designed for factorial experiments was applied with diet, sampling week and sex as factors and bird identifier as the subject.

Wald-type statistic	F	df	*p*-value
Treatment	2.210	1	0.137
Sex	3.117	1	0.078
Week	15.739	3	0.001
Diet x Sex	0.099	1	0.752
Diet x Week	17.160	3	0.001
Sex x Week	4.090	3	0.252
Diet x Sex x Week	5.977	3	0.113

Supplementary Table 2. ASVs affected by nicotine and anabasine diet in the different sampling weeks. Linear discriminant analysis (LDA) effect size (LEfSe) results comparing the control and treatment birds (C/T) after 2, 4 and 7 weeks of treatment. Eighteen ASVs with significant change in relative abundance ($p < 0.05$, LDA effect size >2) are listed (see also Fig. 4).

**ASV ID**	**Week 2**			**Week 4**			**Week 7**			**Taxonomy: Genus (Phylum)**
	C/T	LDA	*p* value	C/T	LDA	*p* value	C/T	LDA	*p* value	
ASV0003	T	3.32		C	4.36	0.0372				*Rothia* (*Alphaproteobacteria*)
ASV0005				C	2.74	0.0109				*Exiguobacterium* (*Firmicutes*)
ASV0007	C	3.30	0.0147							*Klebsiella* (*Gammaproteobacteria*)
ASV0009	T	2.84	0.0061	T	3.63	0.0006	T	3.2	0.0381	*Salmonella* (*Gammaproteobacteria*)
ASV0010	C	2.76	0.0035							*Leuconostoc* (*Firmicutes*)
ASV0012				C	2.74	0.0147				*Acinetobacter* (*Gammaproteobacteria*)
ASV0013							C	2.29	0.0471	*Acinetobacter* (*Gammaproteobacteria*)
ASV0016				T	2.83	0.0381				*Enterococcus* (*Firmicutes*)
ASV0021	C	2.43	0.0471							*Leuconostoc* (*Firmicutes*)
ASV0029							T	2.28	0.0159	Stenotrophomonas (*Gammaproteobacteria*)
ASV0034							C	2.22	0.0339	*Brevibacterium* (*Actinobacteria*)
ASV0040							T	2.35	0.0376	*Chryseobacterium* (*Bacteroidetes*)
ASV0050	T	2.58	0.0367							*Acinetobacter* (*Gammaproteobacteria*)
ASV0057				C	2.74	0.0102				*Streptococcus* (*Firmicutes*)
ASV0071	C	2.98	0.0175							*Empedobacter* (*Bacteroidetes*)
ASV0072	T	2.60	0.0061	T	3.55	0.0376				*Stenotrophomonas* (*Gammaproteobacteria*)
ASV0089				C	3.15	0.0239				*Bifidobacterium* (*Actinobacteria*)
ASV0140				T	3.57	0.0061				*Sphingomonas* (*Alphaproteobacteria*)

Supplementary Table 3. A comparison between the ASVs and the culturable isolate sequences. The culturable isolates that are listed in the table were able to degrade nicotine and anabasine and were 100% identical to the respective ASVs' sequences. See also Figure 5.

Genus	ASV	ASV identity (Genbank BLAST)	Isolates' identity at the species level
Acinetobacter	ASV0013	*Acinetobacter pittii* DSM 21653 strain CIP 70.29 (100%)	*Acinetobacter pittii* (100.0%)
	ASV0019	*Acinetobacter junii* strain ATCC 17908 (100)	*Acinetobacter junii* (99.8%)
Brevibacterium	ASV0034	*Brevibacterium sanguinis* strain CF63 (100%)	*Brevibacterium sanguinis* (99.7%)
Chryseobacterium	ASV0040	*Chryseobacterium gleum* strain NCTC11432 (100%)	*Chryseobacterium gleum* (99.6%)
Delftia	ASV0056	*Delftia lacustris* strain 332 (100%)	*Delftia lacustris* (99.0%)
Exiguobacterium	ASV0005	*Exiguobacterium indicum* strain HHS 31 (100)	*Exiguobacterium indicum* (99.4%)
Klebsiella	ASV0007	*Klebsiella grimontii* strain SB73 (100%)	*Klebsiella grimontii* (99.8%)
Kocuria	ASV0086	*Kocuria palustris* strain TAGA27 (100%)	*Kocuria palustris* (98.5%)
Lactococcus	ASV0001	*Lactococcus lactis* subsp. *Hordniae* strain JCM1 (100%)	*Lactococcus lactis* subsp. *Hordniae* (99.7%)
Methylorubrum	ASV0105	*Methylorubrum populi* BJ001 (100%)	*Methylorubrum populi* (99.1%)
Pseudomonas	ASV0039	*Pseudomonas aeruginosa* DSM50071 (100%)	*Pseudomonas aeruginosa* (99.9%)
Roseomonas	ASV0316	*Roseomonas mucosa* strain MDA5527 (100%)	*Roseomonas mucosa* (99.6%)
Sphingobacterium	ASV0257	*Sphingobacterium spiritivorum* strain NBRC14948 (100%)	*Sphingobacterium spiritivorum* (99.9%)
	ASV0068	*Sphingobacterium multivorum* strain NBRC14947 (100%)	*Sphingobacterium multivorum* (98.9%)
Stenotrophomonas	ASV0117	*Stenotrophomonas maltophilia* strain IAM12423 (100%)	*Stenotrophomonas maltophilia* (99.7%)
	SV0029	*Stenotrophomonas rhizophila* strain e-p10 (100%)	*Stenotrophomonas rhizophila* (99.4%)

Supplementary Table 4. **The relative abundances of the 24 bacterial genera capable of degrading nicotine that were detected in different sampling weeks**. Evidence for nicotine or anabasine degradation properties are either from the current study or from the literature (see also Table 2). W, week; C, control; T, Treatment

Genus	0WC	0WT	2WC	2WT	4WC	4WT	7WC	7WT	References
Achromobacter	0	0	0	0	0	0.000035 ± 3.5E-05	0	0	[1]
Acinetobacter	0.231704 ± 0.0527	0.184708 ± 0.0671	0.023299 ± 0.0038	0.029128 ± 0.0060	0.014617 ± 0.0042	0.011058 ± 0.0048	0.000935 ± 0.0004	0.000546 ± 0.0003	Current study
Arthrobacter	0	0	0	0	0.000033 ± 3.3E-05	0	0	0	[2]
Brevibacterium	0.000057 ± 5.7E-05	0.000055 ± 4.6E-05	0.000253 ± 0.0002	0.000035 ± 3.5E-05	0.000634 ± 0.0003	0.000359 ± 0.0001	0.000849 ± 0.0003	0.000164 ± 6.4E-05	[3]
Brevundimonas	0	0	0	0	0.000190 ± 7.5E-05	0.000338 ± 0.0001	0	0.000026 ± 2.0E-05	[4]
Cellulomonas	0	0.000030 ± 3.0E-05	0	0	0.000075 ± 5.3E-05	0	0	0	[5]
Chryseobacterium	0.000506 ± 0.0005	0	0.000078 ± 7.8E-05	0.000086 ± 8.7E-05	0.000109 ± 7.2E-05	0.000903 ± 0.0007	0	0.000653 ± 0.0004	Current study
Comamonas	0	0	0.000117 ± 5.6E-05	0.000064 ± 3.2E-05	0.000127 ± 8.5E-05	0.000107 ± 7.0E-05	0.000093 ± 6.2E-05	0.000053 ± 4.0E-05	[6]
Delftia	0.000052 ± 5.2E-05	0.000061 ± 6.1E-05	0	0	0	0.000062 ± 6.2E-05	0	0	[7]
Exiguobacterium	0.134430 ± 0.0468	0.152816 ± 0.0741	0.080503 ± 0.0308	0.049391 ± 0.0215	0.006562 ± 0.0023	0.001969 ± 0.0010	0.014405 ± 0.0108	0.000676 ± 0.0004	Current study
Klebsiella	0.000344 ± 0.0003	0.013727 ± 0.0076	0.0374056 ± 0.0131	0.012536 ± 0.0034	0.010750 ± 0.0038	0.026072 ± 0.0075	0.017434 ± 0.0075	0.016650 ± 0.0031	[8]
Kocuria	0	0	0	0	0.000533 ± 0.0004	0.000047 ± 4.7E-05	0.000255 ± 0.0002	0.000038 ± 2.6E-05	Current study
Lactobacillus	0	0	0	0	0.000249 ± 0.0002	0.000061 ± 6.1E-05	0	0	[9]
Lactococcus	0.162420 ± 0.0562	0.073474 ± 0.0421	0.349184 ± 0.0604	0.352911 ± 0.0350	0.143419 ± 0.0355	0.281323 ± 0.0442	0.358921 ± 0.0399	0.495347 ± 0.0449	Current study
Massilia	0	0	0	0	0.000007 ± 7.3E-06	0	0	0	[4]
Methylorubrum	0.000042 ± 4.2E-05	0.000099 ± 8.7E-05	0	0.000005 ± 4.8E-06	0.000446 ± 0.0002	0.000178 ± 0.0001	0	0.000013 ± 1.3E-05	Current study

Paracoccus	0	0	0	0	0.000190 ± 0.0002	0.000070 ± 4.9E-05	0	0	[10]
Pseudomonas	0.013884 ± 0.0072	0.002199 ± 0.0017	0.0014017 ± 0.0004	0.002162 ± 0.0007	0.000773 ± 0.0004	0.010650 ± 0.0094	0.000218 ± 0.0001	0.000076 ± 3.2E-05	[11]
Rhodobacter	0	0	0	0	0	0.000021 ± 2.1E-05	0	0	[12]
Roseomonas	0	0	0	0	0	0.000074 ± 5.6E-05	0	0	Current study
Shinella	0	0	0	0	0.000210 ± 0.0002	0	0	0	[13]
Sphingobacterium	0	0.000007 ± 6.9E-06	0	0	0.000023 ± 1.6E-05	0.001876 ± 0.0018	0	0	[7]
Sphingomonas	0	0	0	0	0.000055 ± 5.5E-05	0.000528 ± 0.0003	0	0	[14]
Stenotrophomonas	0.000677 ± 0.0003	0.003652 ± 0.0029	0.000144 ± 0.0001	0.000737 ± 0.0003	0.000642 ± 0.0002	0.008854 ± 0.0057	0	0.000720 ± 0.0004	[15]

References for Supplementary Table 4.

1. Hylin, J. W. Microbial degradation of nicotine. I. Morphology and physiology of *Achromobacter nicotinophagum* n. sp. *J. Bacteriol*. **76,** 36–40 (1958).
2. Ruan, A., Min, H. & Zhu, W. Studies on biodegradation of nicotine by *Arthrobacter* sp. strain HF-2. *J. Environ. Sci. Heal. - Part B.* **41,** 1159–1170 (2006).
3. Sabourmoghaddam, N., Zakaria, M. P. & Omar, D. Evidence for the microbial degradation of imidacloprid in soils of Cameron highlands. *J. Saudi Soc. Agric. Sci.* **14**, 182–188 (2015).
4. Lei, L., Xia, Z., Liu, X. & Wei, H. L. Occurrence and variability of tobacco rhizosphere and phyllosphere bacterial communities associated with nicotine biodegradation. *Ann. Microbiol.* **65**, 163–173 (2015).
5. Newton, R. P., Jewell, J. N., Geiss, V. L., Knobs, F. & Gravely, L. E. Tobacco with reduced nicotine content due to microbial treatment. United States Patent (19) (1979).
6. Yang, Y. *et al.* Cloning, expression and functional analysis of nicotinate dehydrogenase gene cluster from *Comamonas testosteroni* JA1 that can hydroxylate 3-cyanopyridine. *Biodegradation* **21**, 593–602 (2010).
7. Ma, G. Diversity and phylogenetic analyses of nicotine-degrading bacteria isolated from tobacco plantation soils. *Afr. J. Microbiol. Res.* **6**, 6392–6398 (2012).
8. Ruan, A. & Min, H. Studies on microbiological degradation of tobacco tar. *J. Environ. Sci. Heal.* **40**, 2073–2083 (2005).
9. Chaudhary, N., Qazi, J. I. & Gill, A. Isolation and optimization of tobacco decomposing *Bacillus* and *Lactobacillus* sp. *Caspian J. Env. Sci*. **5**, 45–49 (2007).
10. Qiao, L. & Wang, J. long. Microbial degradation of pyridine by *Paracoccus* sp. isolated from contaminated soil. *J. Hazard. Mater.* **176**, 220–225 (2010).
11. Hu, H. *et al.* Regulatory mechanism of nicotine degradation in *Pseudomonas putida*. *mBio*. **10**, e00602 - 19 (2019).
12. Xia, Z., Lei, L., Zhang, H. Y. & Wei, H. L. Characterization of the ModABC molybdate transport system of *Pseudomonas putida* in nicotine degradation. *Front. Microbiol.* **9**, 3030 (2018).
13. Jiang, H. J., Ma, Y., Qiu, G. J., Wu, F. L. & Chen, S. L. Biodegradation of nicotine by a novel Strain *Shinella* sp. HZN1 isolated from activated sludge. *J. Environ. Sci. Health. B.* **46**, 703–708 (2011).
14. Wang, H., Zhi, X. Y., Qiu, J., Shi, L. & Lu, Z. Chaacterization of a novel nicotine degradation gene cluster ndp in *Sphingomonas melonis* TY and its evolutionary analysis. *Front. Microbiol.* **8**, 337 (2017).
15. Gaekwad, I. & Vinuchurkar, A. S. Isolation and identification of nicotine utilizing bacterial species from tobacco leaves. *Int. Res. J. Pharm.* **9**, 103–106 (2018).
