# Supplementary material for: The effect of toxic pyridine-alkaloid secondary metabolites on the sunbird gut microbiome
Source: NPJ Biofilms Microbiomes. 2020 Nov 13;6:53. doi: 10.1038/s41522-020-00161-9 (PMC7666580; doi:10.1038/s41522-020-00161-9)
Supplement: Supplementary file 2 — Reporting Summary [file 41522_2020_161_MOESM2_ESM.pdf]

nature research

Corresponding author(s): MALKA HALPERN

Last updated by author(s): 2020.07.12

Reporting Summary

Nature Research wishes to improve the reproducibility of the work that we publish. This form provides structure for consistency and transparency in reporting. For further information on Nature Research policies, see our Editorial Policies and the Editorial Policy Checklist.

Statistics

For all statistical analyses, confirm that the following items are present in the figure legend, table legend, main text, or Methods section.

n/a	Confirmed	
☐	☒	The exact sample size (n) for each experimental group/condition, given as a discrete number and unit of measurement
☒	☐	A statement on whether measurements were taken from distinct samples or whether the same sample was measured repeatedly
☐	☒	The statistical test(s) used AND whether they are one- or two-sided<br>*Only common tests should be described solely by name; describe more complex techniques in the Methods section.*
☐	☒	A description of all covariates tested
☒	☐	A description of any assumptions or corrections, such as tests of normality and adjustment for multiple comparisons
☐	☒	A full description of the statistical parameters including central tendency (e.g. means) or other basic estimates (e.g. regression coefficient) AND variation (e.g. standard deviation) or associated estimates of uncertainty (e.g. confidence intervals)
☐	☒	For null hypothesis testing, the test statistic (e.g. F, t, r) with confidence intervals, effect sizes, degrees of freedom and P value noted<br>*Give P values as exact values whenever suitable.*
☒	☐	For Bayesian analysis, information on the choice of priors and Markov chain Monte Carlo settings
☐	☒	For hierarchical and complex designs, identification of the appropriate level for tests and full reporting of outcomes
☒	☐	Estimates of effect sizes (e.g. Cohen's d, Pearson's r), indicating how they were calculated

Our web collection on statistics for biologists contains articles on many of the points above.

Software and code

Policy information about availability of computer code

Data collection	*Provide a description of all commercial, open source and custom code used to collect the data in this study, specifying the version used OR state that no software was used.*
Data analysis	R version 3.6.3

For manuscripts utilizing custom algorithms or software that are central to the research but not yet described in published literature, software must be made available to editors and reviewers. We strongly encourage code deposition in a community repository (e.g. GitHub). See the Nature Research guidelines for submitting code & software for further information.

Data

Policy information about availability of data

All manuscripts must include a data availability statement. This statement should provide the following information, where applicable:

- Accession codes, unique identifiers, or web links for publicly available datasets
- A list of figures that have associated raw data
- A description of any restrictions on data availability

The sequence data from this study are available under the BioProject accession number PRJNA548382 in National Center for Biotechnology Information (NCBI) Sequence Read Archive (https://www.ncbi.nlm.nih.gov/bioproject/). The 16s rRNA sequences are available in the GenBank database under the accession numbers: MK348690–MK348835.

Field-specific reporting

Please select the one below that is the best fit for your research. If you are not sure, read the appropriate sections before making your selection.

☐ Life sciences ☐ Behavioural & social sciences ☒ Ecological, evolutionary & environmental sciences

For a reference copy of the document with all sections, see nature.com/documents/nr-reporting-summary-flat.pdf

Ecological, evolutionary & environmental sciences study design

All studies must disclose on these points even when the disclosure is negative.

Study description	In order to study the effects of pyridine type alkaloids on the gut microbiome of sunbirds we captured sixteen sunbirds and were acclimated to laboratory conditions and fed artificial nectar (Sunbird nectar special formula for Nectariniidae; Aves & Avian, Lot nr IS240718; Reg.nr. NL113333, Raalte, Netherlands) and water, for a period of four weeks. After four weeks of acclimation, the birds were grouped by gender, and then each gender was randomly divided into two groups. The control group (eight birds, four males and four females) was fed the artificial nectar mentioned above, without additional nutrients. The treatment group (eight birds, four males and four females) was fed the same artificial nectar with the addition of nicotine and anabasine in concentrations that naturally occur in N. glauca (0.5 ppm and 5 ppm, respectively 2). Fresh artificial nectar and water were supplied to both groups, daily. Excreta were collected from each of the 16 birds on day zero (before adding nicotine and anabasine to the treatment nectar) and at the end of weeks 2, 4 and 7. The collected excreta are stored at -20 degree and are subjected to DNA isolation, amplification and analysis by illumina analysis.
Research sample	The samples used in this study are excreta samples collected from the sunbirds
Sampling strategy	the sampling size is not calculated as the samples are excreta samples of the sunbirds. But for analysis of samples approximately 1 mL of excreta was used.
Data collection	The sample collection, processing are done by the first author of this article - Mohanraj Gunasekaran. The illumina analysis of samples and their data was collected by the DNA Services Facility, University of Illinois, Chicago. the collected data are analyzed by the authors - Mohanraj Gunasekaran, Yehonatan Sharaby and Maya Lalzar
Timing and spatial scale	The over all study was up to 7 weeks. The first two time space was two weeks intervals and the last one is at 3 weeks intervals. the start of day 0 sample collection was 24.01.2018 and final date was 14.03.2018
Data exclusions	No Data exclusion was done.
Reproducibility	All the computational analysis and statistical analysis are reproducible
Randomization	the 16 birds were grouped by gender, and then each gender was randomly divided into two groups. The control group (eight birds, four males and four females) and the treatment group (eight birds, four males and four females)
Blinding	Blinding was not relevant to our study as its involved just treating birds with alkaloids and collecting their excreta for analysis of their microbiome.

Did the study involve field work? ☐ Yes ☒ No

Reporting for specific materials, systems and methods

We require information from authors about some types of materials, experimental systems and methods used in many studies. Here, indicate whether each material, system or method listed is relevant to your study. If you are not sure if a list item applies to your research, read the appropriate section before selecting a response.

Materials & experimental systems

n/a	Involved in the study	
☒	☐	Antibodies
☒	☐	Eukaryotic cell lines
☒	☐	Palaeontology and archaeology
☐	☒	Animals and other organisms
☒	☐	Human research participants
☒	☐	Clinical data
☒	☐	Dual use research of concern

Methods

n/a	Involved in the study	
☒	☐	ChIP-seq
☒	☐	Flow cytometry
☒	☐	MRI-based neuroimaging

Animals and other organisms

Policy information about studies involving animals; ARRIVE guidelines recommended for reporting animal research

Laboratory animals	*For laboratory animals, report species, strain, sex and age OR state that the study did not involve laboratory animals.*
Wild animals	Cinnyris osea (sunbirds) - both male and female birds was used. The sunbirds are released to the environment after the study completed.
Field-collected samples	In total, 16 adult sunbirds were captured between December 2017 and January 2018 with mist nets and held in captivity for about 12 weeks. Each bird was held in a separate cage in a room with controlled temperature (25 °C) and 12h:12h light:dark conditions. After the experiment ended, the birds were set free.
Ethics oversight	All methods were performed in accordance with relevant guidelines and regulations. Sunbirds were captured in Israel with the permission of the Israel Nature and Parks Authority (permit #2016/41432). All experimental procedures and animal care were approved by the Committee of Animal Experimentation of the University of Haifa (permit #477/16, expiration date September 2020).

Note that full information on the approval of the study protocol must also be provided in the manuscript.
